# Supplementary material for: Microwave Synthesis of Visible-Light-Activated g-C3N4/TiO2 Photocatalysts
Source: Nanomaterials (Basel). 2023 Mar 17;13(6):1090. doi: 10.3390/nano13061090 (PMC10057508; doi:10.3390/nano13061090)
Supplement: Supplementary file 1 [file nanomaterials-13-01090-s001.zip › nanomaterials-2276058-supplementary.pdf]

Supplementary Materials

Microwave Synthesis of Visible-Light-Activated g-C_3N_4/TiO_2 Photocatalysts

Figure S1 shows the BF-TEM image of a thin g-C_3N_4 sheet covered by TiO_2 nanocrystals composing the 30-GCN-T material.

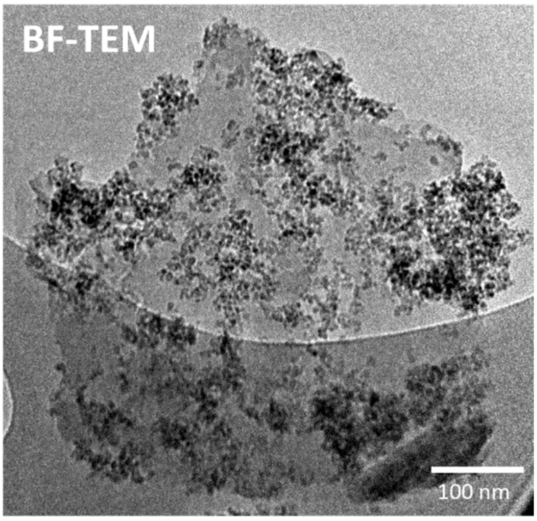

Figure S1. BF-TEM image of the 30-GCN-T material, demonstrating a g-C_3N_4 sheet with TiO_2 nanocrystals.

Figure S2 shows the deconvoluted PL spectra for g-C_3N_4 and 30-GCN-T materials.

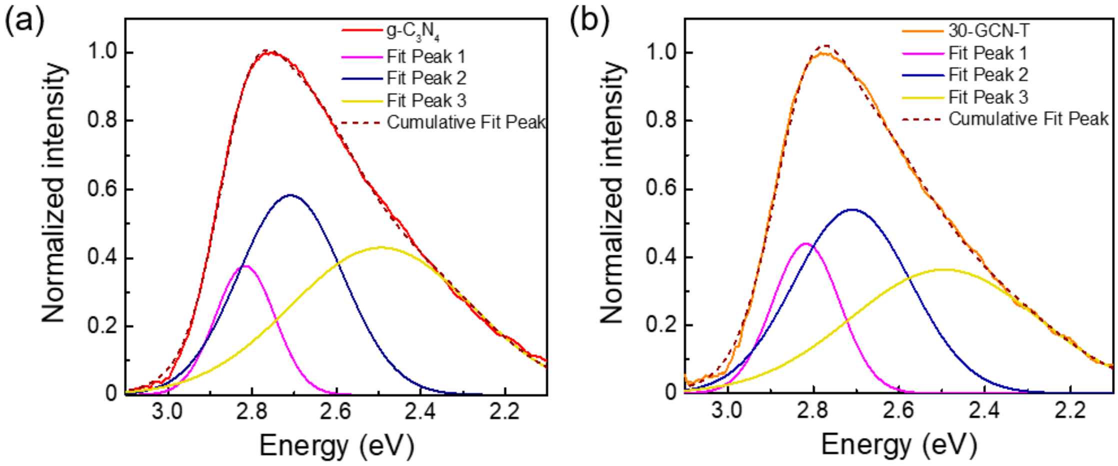

Figure S2. Spectral deconvolution of the broad visible bands into three components for (**a**) g-C_3N_4 and (**b**) 30-GCN-T materials. An adequate fitting was obtained using three Gaussian functions peaked at ~2.817 eV, ~2.709 eV and ~2.494 eV, as described in the main text.

Figure S3 shows the absorbance spectra of the MO solution recorded at different degradation times under solar simulating light and in the presence of each synthesized photocatalyst.

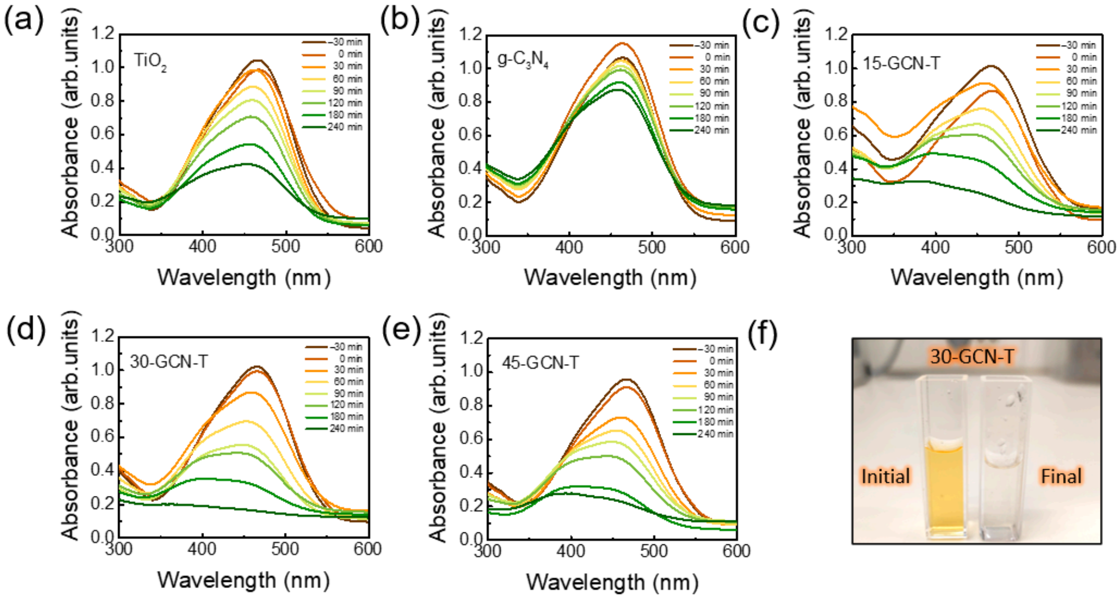

Figure S3. Absorbance spectra of the MO photocatalytic degradation under solar simulating light using the synthesized nanopowders of (**a**) TiO_2, (**b**) $g-C_3N_4$, (**c**) 15-GCN-T, (**d**) 30-GCN-T and (**e**) 45-GCN-T. Photographic image of the MO solution before and after its degradation in 240 min under solar simulating light and in the presence of 30-GCN-T photocatalyst (**f**).
